# Effect of Surface Treatment with Alkaline Agents at Two Different Temperatures on Microshear Bond Strength of Zirconia to Composite Resin

**DOI:** 10.1155/2024/7720286

**Published:** 2024-03-28

**Authors:** Faeze Jamali Zavare, Seyed Reza Khosravani, Moein Sabzivand, Narges Panahandeh

**Affiliations:** ^1^Department of Operative Dentistry, School of Dentistry, Shahid Beheshti University of Medical Sciences, Tehran Postcode: 1983969411, Iran; ^2^School of Dentistry, Shahid Beheshti University of Medical Sciences, Tehran Postcode: 1983969411, Iran

## Abstract

**Background:**

Zirconia, with its excellent mechanical properties, has become a popular choice for esthetic and durable restorations due to the increasing demand of patients. It has overcome most of the limitations of all ceramic restorations. However, bonding to zirconia remains a challenge.

**Objectives:**

This study is aimed at assessing the effect of surface treatment with alkaline agents at two different temperatures on microshear bond strength (*μ*SBS) of zirconia to composite resin.

**Materials and Methods:**

This in vitro, experimental study was conducted on zirconia blocks measuring 2 × 4 × 8 mm. The blocks were sandblasted with alumina powder and randomly assigned to 5 groups (*n* = 16 each). The blocks in groups 1 and 2 underwent surface treatment with sodium hydroxide (NaOH) and groups 3 and 4 with zirconium hydroxide (Zr(OH)4) at room temperature and 70°C. Group 5 served as the control group and did not receive any surface treatment. After the application of bonding agent and its light-curing, composite cylinders in plastic tubes were bonded to the surface of each block and cured. After incubation, they underwent *μ*SBS test. Data were analyzed by one-way ANOVA and Tukey's test (alpha = 0.05).

**Results:**

The *μ*SBS was significantly higher in all intervention groups than that in the control group (*P* < 0.05). The *μ*SBS in Zr(OH)4 groups was significantly higher than that in NaOH groups (*P* < 0.05). The mean *μ*SBS of heated groups was slightly, but not significantly, higher than the corresponding room temperature groups (*P* > 0.05).

**Conclusion:**

Surface treatment of zirconia with NaOH and Zr(OH)4 alkaline agents can increase its *μ*SBS to composite resin; Zr(OH)4 was significantly more effective than NAOH for this purpose, but heating did not have a significant effect on *μ*SBS.

## 1. Introduction

Dental ceramics are extensively used for indirect tooth restorations [1, 2]. Zirconia is highly popular in dentistry due to its unique properties such as high biocompatibility and optimal esthetics. Its mechanical properties resemble those of metals while its color ideally mimics the tooth color [3–5]. It is commonly used for the fabrication of prosthetic crowns, implant abutments, frameworks, intracanal posts, orthodontic brackets, etc. [1, 2, 6]. Although zirconia has been used for dental applications since 1960, the advent of computer-aided design/computer-aided manufacturing technology revolutionized its applications and further added to its popularity [6]. Zirconia ceramics have the highest fracture resistance and toughness among all ceramic materials [7]. Zirconia can have a flexural strength as high as 700 to 1200 MPa, which exceeds the maximum load applied to the teeth during mastication. Also, it has a fracture resistance of 2000 N, which is twice the fracture resistance of alumina ceramic and thrice the fracture resistance of lithium disilicate ceramic. Such unique properties of zirconia are attributed to its microstructural phase transformation under tension, such that tetragonal to monoclinic phase transformation prevents propagation of small cracks [8].

Nonetheless, the long-term clinical success of zirconia ceramics depends on their stable bonding to the substrate including the tooth structure and composite resin [7]. Evidence shows that the conventional techniques of adhesive cementation such as acid etching of ceramic surface with hydrofluoric acid are not suitable for zirconia ceramics and do not create a sufficiently strong bond. The reason can be attributed to the silica-free structure of zirconia, which prevents the formation of siloxane network with silane [6, 8–10].

Chemomechanical retention is required for a stable bond between ceramic and composite resin. Different surface treatments have been proposed to enhance bonding to zirconia, such as sandblasting, tribochemical silicoating, and laser irradiation [1, 2, 10, 11]. Sandblasting creates a porous surface and increases the bonding surface area and micromechanical retention [1]. Also, evidence shows that sandblasting of zirconia can increase the bond strength of zirconia under mechanical tensions, prevent crack initiation and progression, and increase the durability of restoration [1, 2]. Arami et al. [2] demonstrated that alumina sandblasting significantly increased the microshear bond strength (*μ*SBS) of zirconia to composite resin and was more effective than Er:YAG and Nd:YAG laser irradiation.

Chemical surface treatments have also been suggested along with mechanical surface treatments to further enhance the bond strength to zirconia [10, 12, 13] such as application of bonding agents containing hydrophobic phosphate monomers, e.g., 10-methacryloyloxydecyldihydrogen¬phosphate (10-MDP) [8, 10, 13, 14]. It has a hydrophobic methacrylate group at one end, which can bond to methacrylate-based restorations and cements, and has a polar phosphate group at the other end, which can bond to zirconia [1, 10, 13, 15]. Primers containing 10-MDP create a reactive surface between zirconia and composite resin and enhance the *μ*SBS of zirconia to composite [13, 14]. Also, evidence shows that using 10-MDP-containing adhesive monomers in an alkaline environment can increase the bond strength of zirconia to composite resin [10, 13, 16]. Due to nonpolarity of zirconia surface, it has low chemical reactivity [16]. By creating an alkaline environment and by increasing the (OH)- groups on the zirconia surface and isolation of H+ ions, surface wettability increases, and a higher number of Zr-O-P structures are formed [10]. Qian et al. [13] used four different alkaline agents of sodium hydroxide (NaOH), calcium hydroxide (Ca(OH)2), magnesium oxide (MgO), and zirconium oxide (Zr(OH)4) to enhance the bonding of zirconia to composite through 10-MDP and reported that all of them significantly increased the *μ*SBS of zirconia to composite resin. Also, Zr(OH)4 yielded the highest *μ*SBS while NaOH yielded the highest thermodynamic strength.

Considering the increasing applications of zirconia ceramics in dentistry, further studies are imperative to find an ideal technique to enhance the bond strength of zirconia to composite resin, and still, different bonding systems and environmental conditions need to be tested for this purpose [6]. Since the conduction of chemical reactions at higher temperatures may improve the reaction efficiency, this study is aimed at assessing the effect of surface treatment of zirconia with NaOH and Zr(OH)2 at two different temperatures on *μ*SBS of zirconia to composite resins.

## 2. Materials and Methods

The protocol of this study was approved by the ethics committee of Shahid Beheshti University of Medical Sciences (IR.SBMU.DRC.REC.1399.094). This in vitro, experimental study was conducted on 80 cubic zirconia blocks (DDBio ZX2; Dental Direkt,106-108, 32139 Spenge, Germany) with no cracks or voids, measuring 2 × 4 × 8 mm. The sample size was calculated to be 80 specimens assuming alpha = 0.05, beta = 0.2, and study power of 80%. The specimen surfaces were polished with 600-grit silicon carbide abrasive papers to create standardized smooth surfaces and were then dried with air spray. They were then sintered, cleaned with distilled water in an ultrasonic bath, and dried in a desiccator for 24 hours [16]. They were then sandblasted with 110 *μ*m alumina (Al2O3) particles from a 1 cm distance for 10 seconds and were then randomly assigned to 5 groups, each containing 16 zirconia blocks, as follows:

- Group 1. Specimens in this group were immersed in 1 molar NaOH solution with a pH of 12 in a stirrer at 70°C for 24 hours and were then completely dried in a desiccator. Next, G-Premio Bond (GC Dental Products Co., Japan) was applied on the specimen surface by a microbrush for 10 seconds. After 20 seconds, the bonding agent was air-thinned with oil-free air spray for 5 seconds as instructed by the manufacturer and light cured at 1 mm distance for 20 seconds with a light intensity of 550 mW/cm^2^
- Group 2. Specimens in this group were immersed in 1 molar NaOH solution with a pH of 12 in a stirrer at room temperature for 24 hours and were then completely dried in a desiccator. The rest of the procedure was similar to group 1
- Group 3. Specimens in this group were immersed in Zr(OH)4 solution with a pH of 12 in a stirrer at 70°C for 24 hours and were then completely dried in a desiccator. The rest of the procedure was similar to group 1
- Group 4. Specimens in this group were immersed in Zr(OH)4 solution with a pH of 12 in a stirrer at room temperature for 24 hours and were then completely dried in a desiccator. The rest of the procedure was similar to group 1
- Group 5. This group served as the control group and did not receive any surface treatment. The surface of specimens was rinsed with distilled water and dried with a desiccator. The bonding procedure was then performed as explained for group 1

Transparent plastic Tygon tubes with an internal diameter of 0.7 mm and 1 mm height were then filled with Z250 composite resin (3M ESPE, St. Paul, MN, USA) and placed on the surface of zirconia specimens (one per each specimen). They were then cured for 40 seconds from each side and placed at room temperature for 1 hour. The plastic tubes were then separated from the composite cylinders by a surgical scalpel, and the specimens were incubated at 37°C and 100% humidity for 24 hours. Next, they were dried in a desiccator for 1 hour and underwent *μ*SBS test in a microtensile tester (Zwick Roell Z020) at a crosshead speed of 0.5 mm/minute. The obtained value in Newtons was divided by the cross-sectional area of composite cylinder in square millimeters to obtain the *μ*SBS value in megapascals (MPa).

Table 1 presents the composition of materials used in this study.

### 2.1. Statistical Analysis

Data were analyzed using SPSS version 26. The normal distribution of data was confirmed by the Kolmogorov-Smirnov test. Thus, data were analyzed by one-way ANOVA. Pairwise comparisons were conducted by Tukey's test. The level of significance was set at 0.05.

## 3. Results

Table 2 presents the mean *μ*SBS of the study groups. As shown, the highest *μ*SBS was recorded in Zr(OH)4 plus heating group and the lowest in the control group. A significant difference was noted in *μ*SBS among the study groups (*P* < 0.05). Thus, pairwise comparisons were carried out (Table 3), which revealed that the *μ*SBS was significantly higher in all intervention groups than that in the control group (*P* < 0.05). The *μ*SBS in Zr(OH)4 groups was significantly higher than that in NaOH groups (*P* < 0.05). The mean *μ*SBS in Zr(OH)4 plus heating group was slightly, but not significantly, higher than that in the room temperature group (*P* > 0.05). The same result was obtained for NaOH groups with and without heating.

## 4. Discussion

This study assessed the effect of surface treatment with NaOH and Zr(OH)2 at two different temperatures on *μ*SBS of zirconia to composite resin. The results showed significantly higher *μ*SBS in NaOH and Zr(OH)4 groups compared with the control group. Xie et al. [17] measured the *μ*SBS of zirconia blocks to composite after bonding under acidic, neutral, and alkaline conditions. They showed significantly higher *μ*SBS of alkaline groups, and the lowest *μ*SBS was found in specimens subjected to acidic pH before the bonding procedure. Other studies also showed the positive efficacy of surface treatment of zirconia with alkaline agents for enhancement of *μ*SBS to composite resin [10, 16]. Electron microscopic observations have shown that acidifying or alkalizing the zirconia surface does not change its surface roughness; thus, the increase in *μ*SBS cannot be attributed to the enhancement of micromechanical retention following alkalization of zirconia surface [17]. It appears that nonpolarity of zirconia surface is responsible for its low chemical reactivity [16]. Under normal conditions, a large space is present between the zirconium atoms on the zirconia surface, and the distance between free electrons is too large to allow their engagement in bonding [17]. However, alkalizing the surface increases the number of OH groups on the zirconia surface and isolates H+ ions; resultantly, surface wettability increases, and a greater number of Zr-O-P structures are formed. This phenomenon may explain the enhancement of SBS of zirconia treated with MDP to composite resin [10, 13, 17]. The increase in SBS is not merely due to pH rise, and the size of particles in alkaline suspension, type of alkaline agent, and durability of these particles on the surface of treated zirconia are also important. Qian et al. [13] indicated that although NaOH and Ca(OH)2 had a higher pH than other alkaline agents, the SBS of specimens treated with MgO and Zr(OH)4 was higher due to smaller size and higher durability of MgO and Zr(OH)4 particles. Studies recommend using nanosize particles to increase their durability and adhesion, and subsequently their effectiveness for enhancement of bond strength. Nanoparticles in these suspensions adhere to the zirconia surface after drying through van der Waals forces [18, 19].

In the present study, the mean *μ*SBS of zirconia specimens treated with Zr(OH)4 was significantly higher than that of specimens treated with NaOH. Similarly, Qian et al. [13] reported the highest SBS in the Zr(OH)4 group. In their study, the concentration of NaOH and Zr(OH)4 used for surface treatment of zirconia was the same. Resultantly, their pH values were different, such that 1 molar solution of NaOH had a higher pH than Zr(OH)4. According to thermodynamic calculations, they expected a higher SBS in the NaOH group while the SBS was found to be higher in the Zr(OH)4 group. In the present study, the pH of NaOH and Zr(OH)4 solutions was the same. It appears that under in vitro conditions, the type of material has a more profound effect on SBS than its pH. The reason may be that Zr(OH)4 coatings have higher advantages for zirconia ceramic restorations. Also, alkaline spots formed on the zirconia surface by Zr(OH)4 were stable under humid conditions while alkaline spots formed by NaOH were unstable in humid conditions [13]. Thus, it appears that in a humid oral environment, Zr(OH)4 forms a stronger bond than NaOH. Previous studies showed that although NaOH coating can significantly increase the bond strength of MDP to zirconia, it cannot prevent microleakage through the resin-zirconia interface in a humid oral environment. Humidity results in dissolution of water-soluble alkaline coatings such as NaOH, while Zr(OH)4 is not water-soluble and has a higher durability in humid environments [20, 21].

The present study also assessed the effect of heating on *μ*SBS of zirconia specimens, and the results showed that although heating at 70° increased the *μ*SBS, this increase was not significant. Lack of a significant increase in *μ*SBS by heating may be due to low heating temperature [22, 23]. Komine et al. [22] evaluated the effect of heating (110°C) on *μ*SBS of zirconia to composite and reported an increase in *μ*SBS in heated groups. The reason may be that sandblasting results in tetragonal to monoclinic phase transformation in zirconia, which decreases the bond strength to composite resin. However, heating reverses this phase transformation and increases the *μ*SBS as such [23].

In vitro design and lack of simulation of clinical setting by thermocycling or water storage were the main limitations of this study, which limit the generalization of results to the clinical setting. Future studies are recommended on *μ*SBS of zirconia treated with different alkaline agents at different concentrations and with different application times. Also, thermocycling should be performed in future studies to better simulate the clinical setting.

## 5. Conclusions

Surface treatment with NaOH and Zr(OH)4 alkaline agents significantly increased the *μ*SBS of zirconia to composite resin, and Zr(OH)4 was significantly more effective than NAOH for this purpose. Although heating increased the *μ*SBS, this increase was not statistically significant.

## Figures and Tables

**Table 1:** Composition of materials used in this study.

Material	Lot number	Symbol	pH	Manufacturer	Composition
Zirconium dioxide		DDBio ZX2		Dental Direkt, 106-108, 32139 Spenge, Germany	ZrO2 + HfO_2_+Y_2_O_3_ (≥99), Y_2_O_3_(<6), Al_2_O_3_, (≤0.15), other oxides (<1.0)
Filtek™ Z250 Universal Restorative	9456914	Z250		3 M ESPE, MN, USA	Bis-GMA, UDMA, Bis-EMA, filler (0.01-3.5 *μ*m)
G-Premio Bond	2212081	GP	1.5	GC DENTAL PRODUCTS CORP, Japan	10-MDP, 4-Methacryloxyethyl trimellitic anhydride, dimethacrylate monomer, distilled water, acetone, photo initiators, fine silica powder
Zirconium hydroxide			12	sigma, Germany	Zr(OH)4
Sodium hydroxide			12	Merck, Germany	NaOH

**Table 2:** Mean *μ*SBS (MPa) of the study groups (*n* = 16 each).

Group	Definition	Mean (SD) Microshear bond strength (MPa)
1	NaOH+ heat	17.94 (±1.10)
2	NaOH	17.4 (±1.34)
3	Zr(OH)4+ heat	22.00 (±1.72)
4	Zr(OH)4	21.67 (±1.81)
5	Control	11.64 (±1.57)

SD: standard deviation.

**Table 3:** Pairwise comparisons of the groups regarding *μ*SBS.

Group (I)	Group (J)	Mean difference	*P* value
Control	NaOH	-5.75	<0.001
	NaOH+ heat	-6.30	<0.001
	Zr(OH)_4_	-10.03	<0.001
	Zr(OH)+ heat	-10.35	<0.001

NaOH	Control	5.75	<0.001
	NaOH+ heat	-.0.54	0.854
	Zr(OH)_4_	-4.27	<0.001
	Zr(OH)+ heat	-4.60	<0.001

NaOH+ heat	Control	6.30	<0.001
	NaOH	0.54	0.854
	Zr(OH)_4_	-3.73	<0.001
	Zr(OH)_4_+ heat	-4.05	<0.001

Zr(OH)4	Control	10.03	<0.001
	NaOH	4.27	<0.001
	NaOH+ heat	3.73	<0.001
	Zr(OH)_4_+ heat	-.0.32	0.975

Zr(OH)+ heat	Control	10.35	<0.001
	NaOH	4.60	<0.001
	NaOH+ heat	4.05	<0.001
	Zr(OH)_4_	0.32	0.975

## Data Availability

The data used to support the findings of this study are available from the corresponding author upon request.
